# Genetic rescue of Florida panthers reduced homozygosity but did not swamp ancestral genotypes

**DOI:** 10.1073/pnas.2410945122

**Published:** 2025-07-28

**Authors:** Diana Aguilar-Gómez, Lin Yuan, Yulin Zhang, Alexander Ochoa, Melanie Culver, Robert R. Fitak, Dave Onorato, Kirk E. Lohmueller, Rasmus Nielsen

**Affiliations:** ^a^Department of Ecology and Evolutionary Biology, University of California, Los Angeles, CA 90095; ^b^Center for Computational Biology, College of Computing, Data Science and Society, University of California, Berkeley, CA 94720; ^c^Cell and Molecular Biology Programme, School of Life Sciences, The Chinese University of Hong Kong, Hong Kong, Special Administrative Region of China; ^d^Department of Ecology & Evolutionary Biology, Yale University, New Haven, CT 06520; ^e^U.S. Geological Survey, Arizona Cooperative Fish and Wildlife Research Unit, University of Arizona, Tucson, AZ 85721; ^f^School of Natural Resources and the Environment, University of Arizona, Tucson, AZ 85721; ^g^Department of Biology, Genomics and Bioinformatics Cluster, University of Central Florida, Orlando, FL 32816; ^h^Fish and Wildlife Research Institute, Florida Fish and Wildlife Conservation Commission, Naples, FL 34114; ^i^Department of Human Genetics, David Geffen School of Medicine, University of California, Los Angeles, CA 90095

**Keywords:** genetic rescue, puma, conservation genomics, genetic swamping, florida panthers

## Abstract

Pumas (*Puma concolor*) occupy a vast geographical range spanning from Canada to Argentina. Due to urbanization and unregulated hunting, pumas in Florida, known as panthers, are the only breeding population east of the Mississippi River. In the 1990s, Florida panthers numbered <30 individuals suffering from inbreeding depression. In 1995, eight pumas from Texas were translocated into southern Florida to mitigate the effects of isolation. This translocation reduced inbreeding depression and increased population size. While genetic rescue is often suggested as a means of ameliorating the effects of small population size, the underlying genetic mechanism and its long-term efficacy remain understudied. We sequenced the genomes of posttranslocation Florida panthers (PTFPs) to elucidate the genomic consequences of genetic rescue. We inferred local ancestry across the genomes of PTFPs and found that no regions have been entirely replaced by Texas ancestry, discarding the possibility of genetic swamping. Furthermore, the beneficial effects of the translocation were likely caused by a reduction in homozygosity, alleviating recessive deleterious load, rather than by a reduction in the number of deleterious variants. We did not find evidence that selection has favored replacement of original Florida DNA with Texas DNA in any systematic fashion. Using simulations, we found that heterozygosity increased in the long-term compared to a no translocation scenario; however, the effects on fitness are more transient. Our findings hold significant implications not only for the management of Florida's panther population, but also for informing strategies for genetic rescue in other wild, inbred populations encompassing broader conservation efforts.

## Significance

In the mid 1990s, Florida panthers were at risk of extinction due to isolation and habitat loss with the population numbering less than 30 individuals, many of whom exhibited morphologic and genetic correlates of inbreeding. In 1995, eight female pumas from Texas were translocated to Florida to attempt “genetic rescue.” We show that the rescue was successful with observed improvements due to increased heterozygosity rather than reduction in the number of deleterious variants. Further, our analysis shows that the Florida genetic ancestry was not completely replaced, thus allying fears that rescue leads to extinction by replacement. We demonstrate that knowledge gained from speciation science can be applied to conservation action via the mechanism of genetic rescue from appropriately diverged populations.

Pumas (*Puma concolor*) are large carnivores that are distributed across a vast range stretching from the Canadian Province of British Columbia to the Chilean Strait of Magellan (1). Despite the extensive geographic distribution of pumas across the Americas, the subspecies of pumas in Florida (*P. concolor coryi*) (hereafter Florida panthers) remain the only viable population of pumas east of the Mississippi River, United States. The decline of panthers was primarily driven by unregulated hunting and fragmentation of habitat due to wide-scale urbanization in the region (2). By the early 1990s, Florida panthers existed as a small, isolated population of <30 individuals inhabiting Big Cypress National Preserve, Everglades National Park, and surrounding hardwood swamps and prairies in southern Florida (3). During this time, panthers confined to the Everglades displayed a mixture of native (i.e., canonical) and Central American (Panama-Costa Rica) ancestries resulting from poorly documented releases of captive-bred pumas in the area between 1956 and 1966 (4–9). In general, however, due to accrued effects of genetic drift and inbreeding, Florida panthers presented a depauperate canonical genetic variation and a high rate of developmental (e.g., atrial septal defects, kinked tails), reproductive (e.g., cryptorchidism, spermatozoal defects), and immunological (i.e., susceptibility to different biological agents) impairments (5, 10). Given these factors, population viability analyses projected the extinction of Florida panthers within 25 to 40 y absent immigration from other puma populations (11).

In 1995, eight female pumas from Texas were translocated into southern Florida as part of a genetic rescue plan to alleviate the occurrence of traits associated with inbreeding depression that would hopefully lead to an increase in abundance of the panther population (2, 12). In subsequent generations, five of these Texas pumas were documented to have produced at least 20 admixed Florida panthers, which collectively manifested an enhanced fitness relative to prerescue Florida panthers (13). For instance, van de Kerk et al. (14) found a positive correlation between kitten survival and their degree of Texas ancestry; a similar trend was noted for survival among adult and subadult age classes in Florida panthers. Moreover, at the genomic level, F_1_ panthers exhibited a nearly threefold increase in observed heterozygosity with respect to the previous, canonical Florida generation (7, 15). This genetic rescue project also had positive long-term effects on Florida panther fitness, as exemplified by the declines in proportion of individuals examined that exhibited cryptorchidism, atrial septal defects, and abnormal sperm when comparing cohorts of panthers born prior to genetic rescue versus admixed panthers born postgenetic rescue (16). As of 2023, the Florida panther population size is believed to range between 120 and 230 adults and subadults (17).

The benefits of translocating individuals with the intent of genetic rescue have, at times, been controversial (18–20). One potential benefit is the introduction of new genetic variation into populations affected by accumulation of (primarily additive) deleterious variants due to reduced population size. If a substantial number of deleterious variants have gone to fixation during a bottleneck, introducing new chromosomes that do not carry these variants can serve as a substrate for selection removing deleterious variants. This in turn may provide substantial gains in fitness after recovery from a bottleneck (21). Another potential benefit is the masking of recessive deleterious alleles due to increased heterozygosity after genetic rescue (22). This effect does not require selection to act, will be effective immediately after genetic rescue, even in the absence of a population size recovery, but requires that a substantial proportion of the original population is replaced by the donor population (23, 24). Hitherto, the exact genomic mechanism causing an increase in fitness after genetic rescue remains unknown.

Potential drawbacks of genetic rescue include the possibility of genomic swamping, i.e., the replacement of the local DNA by DNA from the donor population that may eliminate local adaptive genetic variation (13, 25, 26). This will be a particular risk in systems where individuals from the donor population carry substantially fewer deleterious alleles so that selection will strongly favor the donor population chromosomes (23, 24, 27). Another potential drawback is the possibility the donor population will introduce new deleterious variants (19, 20). If the rescued population has had a small population size for a long period of time, it may have purged recessive deleterious variants, and the introduction of new genetic variation might actually have an overall detrimental effect. In the case of Florida panthers, this is likely not an issue since it is well documented that the population decline was associated with recent human activity (2). However, Ochoa et al. (15) described an increase in the number of heterozygotes carrying deleterious alleles in the F1 generation compared to the pretranslocation Florida panthers. Epistatic interactions between alleles from the donor and the rescued populations may also lead to reductions in fitness (28). While it is clear that the genetic rescue in Florida panthers increased fitness, the degree that it may also have caused genetic swamping is unclear. In this study, our goal is to understand the genomic effects of the genetic rescue on heterozygosity and deleterious variation and determine whether genetic swamping occurred.

## Results

### Genetic Structure of Puma Populations.

**Fig. 1.**
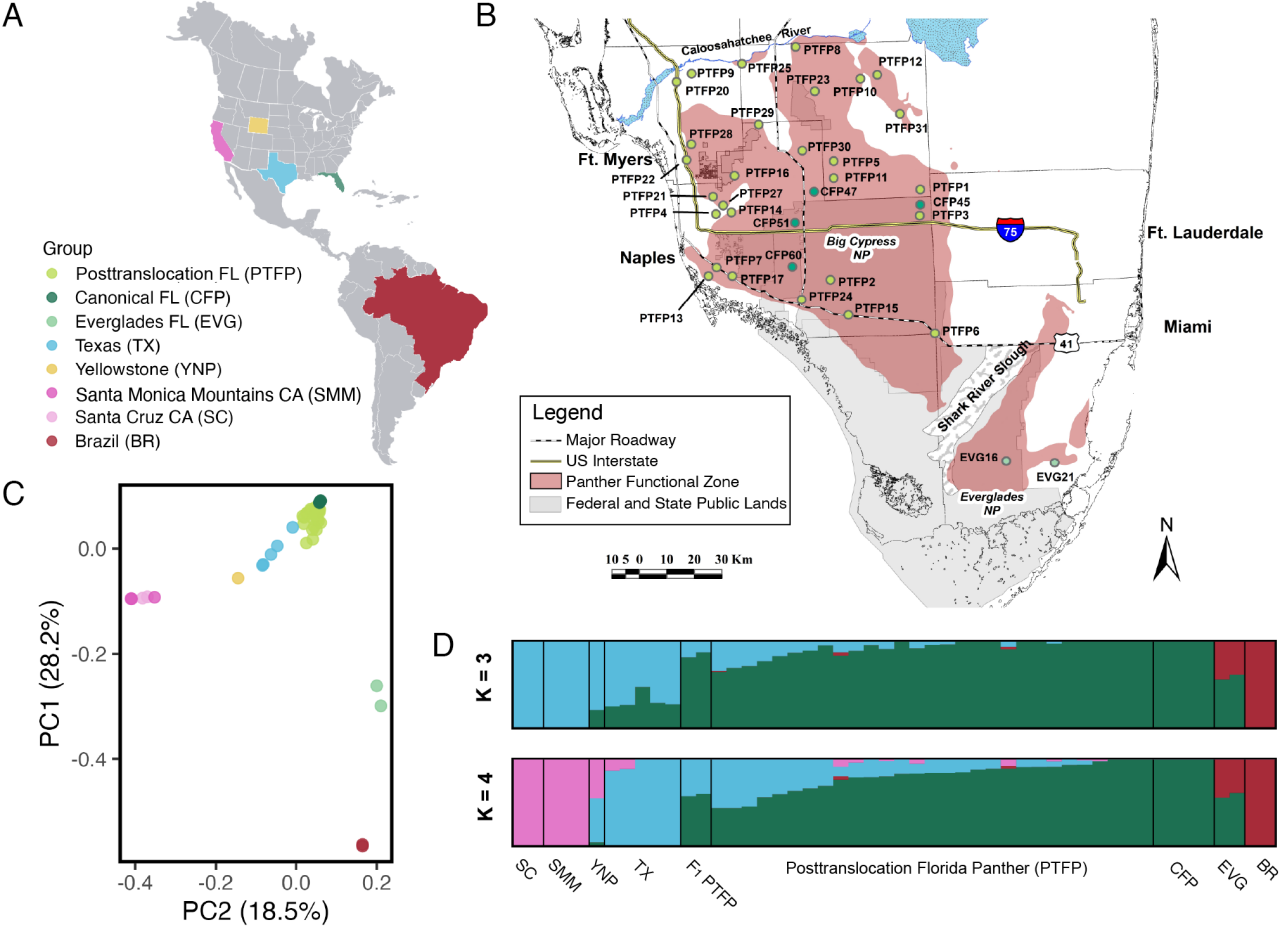
Sampling locations and population structure of pumas in the Americas. (*A*) Map of the sampling regions and code used for the different groups analyzed. (*B*) Collection locations for Florida panther (*P. concolor coryi*) samples in South Florida, USA. The Panther Functional Zone comprises suitable habitat that supports the breeding population of panthers in South Florida from the Caloosahatchee River south to Everglades National Park (30). Three PTFPs are not shown in this map, as they were collected north of this zone. (*C*) Principal component analysis of puma SNPs with a 5% minor allele frequency filter. (*D*) OHANA unsupervised ancestry component analysis, specifying k = 3, 4.

To assess the genomic consequences of genetic rescue via translocation, we generated whole genome sequence data from 29 posttranslocation Florida panthers (PTFPs) (29) (Fig. 1*B*; also see *Materials and Methods* and Dataset S1). To contextualize our findings within the species, we combined these data with publicly available whole-genome sequence data from two previous studies (7, 8) (Fig. 1*A*). We included two individuals from Brazil (BR); five individuals from California, including three from the Santa Monica Mountains (SMM) and two from Santa Cruz (SC); and one from Yellowstone (YNP). Texas (TX) samples comprise the five individuals who were introduced in Florida for genetic rescue in 1995 and successfully mated. We also included two samples from Everglades (EVG) panthers with mixed ancestry (previously noted) and four canonical Florida panthers (CFP). In addition to our 29 PTFP samples, we included two F_1_ (CFPxTX) PTFP individuals (7).

We mapped all reads to the reference genome of *Puma yagouaroundi*, and after filtering and applying a 5% minor allele frequency filter (*Materials and Methods*), we performed Principal Component Analysis on the genetic data (Fig. 1*C*) to assess patterns of population structure. PC1 separates North and South American populations, with EVG clustering in the center, consistent with the known introduction of captive pumas from Central America in that area (4–9). PC2 separates pumas across North America from West to East, with California at the left, followed by Yellowstone, Texas, and Florida at the right. The PTFP samples fall between CFP and TX, as expected.

To further explore genetic structure, we used OHANA (31) to estimate the admixture proportions assuming a standard admixture model in each individual, specifying different numbers of ancestry components (k), while using genotype likelihoods to take uncertainty in genotyping into account. Specifying k = 3, we obtained one component for Central and South America, and two components for North America, separating the populations in West (California) and East (Florida) and some populations being a mix of Eastern and Western ancestral puma components (Texas and Yellowstone) (Fig. 1*D*). When k = 4, California and Texas separate and each are assigned their own component.

### Heterozygosity and Inbreeding After Translocation.

**Fig. 2.**
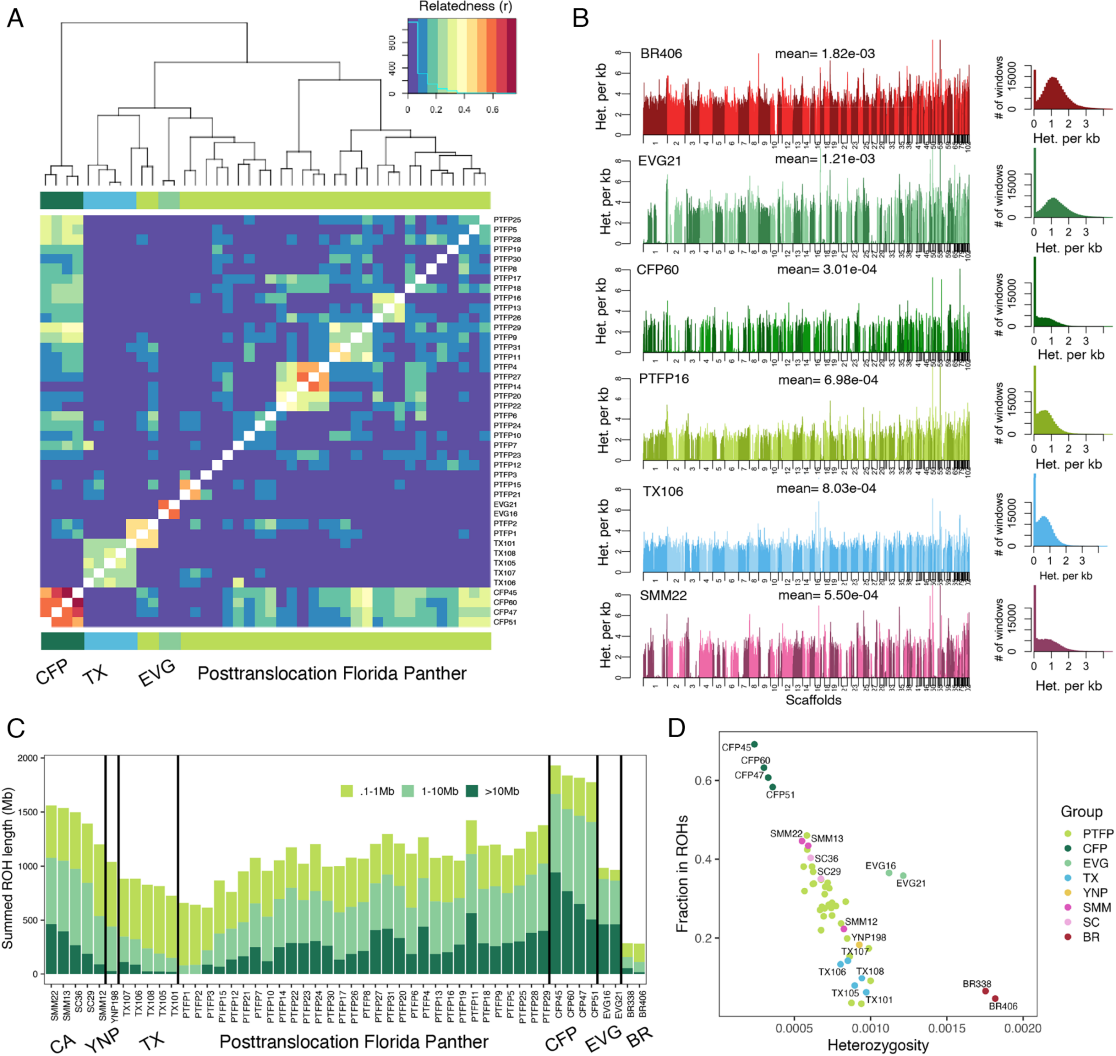
Relatedness, heterozygosity, and ROHs. (*A*) Relatedness matrix of populations in Florida and Texas. (*B*) Each row shows on the left the genome-wide heterozygosity of one representative individual per population (window size = 100 Kb, step = 10 Kb) and on the right a histogram of the distribution of heterozygosity across windows. From top to bottom, the individuals are Brazil (BR406), Everglades (EVG21), canonical Florida panther (CFP60), posttranslocation Florida panther (PTFP16), Texas (TX106), and Santa Monica Mountains (SMM22). Each plot is labeled with the sample name and the mean genome-wide heterozygosity. (*C*) Distribution of runs of homozygosity (ROHs) in each individual, divided into three bins: short 0.1 to 1 Mb, medium 1 to 10 Mb, and long >10 Mb. Vertical panels separate by region: California (CA), Yellowstone (YNP), Texas (TX), posttranslocation Florida panther (PTFP), canonical Florida panther (CFP), Everglades (EVG), Brazil (BR). (*D*) Scatter plot of the fraction of the genome in ROHs (>1 Mb) vs. heterozygosity per individual.

We analyzed genetic relatedness among the pumas using ngsRelate (32), which uses genotype likelihoods and accounts for inbreeding within a population. Before the translocation, CFPs had inferred relatedness values ranging between 0.44 and 0.72, with an average of 0.57, which is similar to the level of relatedness expected for siblings or parent–offspring in outbred populations (Fig. 2*A* and Dataset S2). After the translocation, the relatedness of PTFP individuals decreased to an average of 0.03 (min = 0, max = 0.56).

We calculated heterozygosity in sliding windows across the genome (size = 100 kb, step = 10 kb). The CFPs and Santa Monica Mountains (SMM) pumas have more genomic regions with zero heterozygosity than other groups we sampled (Fig. 2*B*). We next searched for ROHs of different lengths (short 0.1 to 1 Mb, medium 1 to 10 Mb, and long >10 Mb) in each genome and calculated the fraction of the genome in ROHs (>1 Mb; Fig. 2*C*). We found that CFP individuals have the greatest number of long ROHs (30% of their genome is in ROHs >10 Mb). In contrast, Texas (TX) individuals have on average 2% of their genome in long ROHs. The main reason that TX individuals were chosen to be introduced into Florida in 1995, was the historical gene flow suspected between these populations (2) and the lack of morphological defects in TX, which seemed to indicate a genetically healthy population (12). At the time, genomic technologies were not available. Genomic sequencing confirmed that TX and Yellowstone (YNP) have the highest heterozygosity among the North America samples included in this study (π_TX_ = 0.00089, π_YNP_ = 0.00093), excluding Everglades (EVG), which will be discussed below. Second only to individuals from Brazil (BR), TX individuals have the lowest fraction of their genome in ROHs (Fig. 2*D*), with most of it in small ROHs (Fig. 2*C*). Individuals from BR are the least inbred, having the highest heterozygosity (0.00178) and only 5% of their genome in ROHs. The average heterozygosity in EVG pumas (0.00117) is more than three times greater than that in CFP pumas (0.00031). However, EVG pumas have a large proportion of long ROHs (19% of their genome in ROHs >10 Mb) and more than 35% of their genome in ROHs (Fig. 2*D*). In the case of the PTFPs, heterozygosity increased (to 0.00073) on average more than 2× what it was before genetic rescue. The average fraction of the genome in ROHs was reduced to less than half the levels pregenetic rescue (CFP = 63%, PTFP = 28%) (Fig. 2*D*), particularly reducing long ROHs (CFP = 30%, PTFP = 11%) (Fig. 2*C*). Our results (Fig. 2*D*) match the findings of Saremi et al. (8), but we expand those results to include individuals from Texas, posttranslocation Florida, and additional individuals from California, Everglades, and canonical Florida absent from that study.

### Preservation of Local Ancestry and Heterozygosity.

**Fig. 3.**
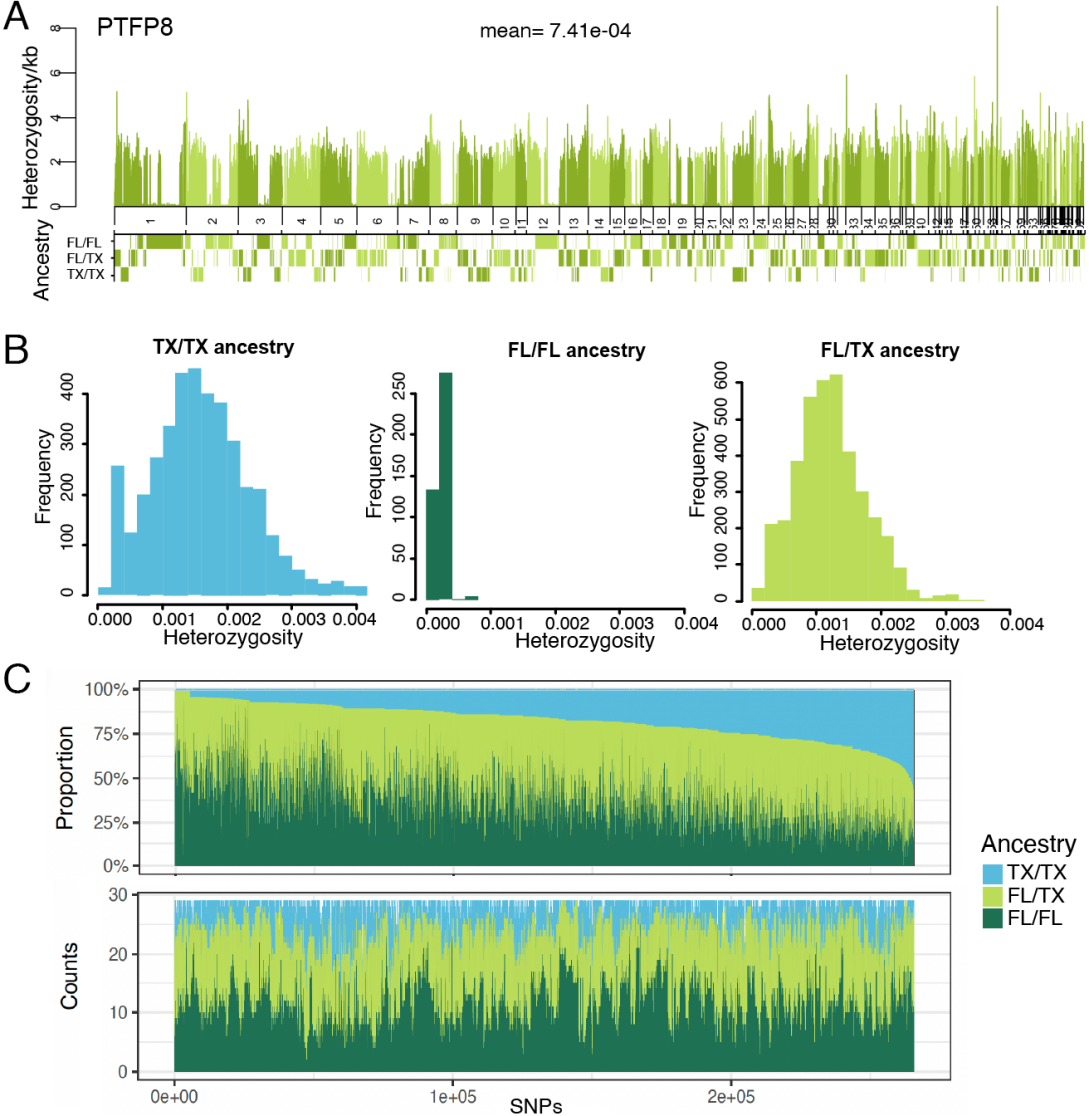
Local ancestry and heterozygosity. (*A*) Genome-wide heterozygosity per kb of a posttranslocation FL panther and the inferred ancestry aligned by scaffold. The plot is labeled with the sample name and the mean genome-wide heterozygosity for that individual. (*B*) Histogram of heterozygosity of windows that correspond to each of the inferred ancestries in the same individual (PTFP8). TX: Texas, FL: Florida. (*C*) *Top*: sorted proportion of individuals that have each of the ancestry inferences. *Bottom*: counts of individuals with each ancestry. SNPs are sorted by position in the scaffold. F_1_ samples were excluded from these plots because they are FL/TX everywhere.

To evaluate local ancestry along the genomes of PTFPs, we used ancestryHMM (33) to identify regions of original Texas (TX) and Florida (FL) ancestry. In contrast to the ancestry component analysis using OHANA (Fig. 1*D*), ancestryHMM infers all PTFP to be admixed, with 24 to 61% TX ancestry (*SI Appendix*, Table S1). Within each individual, we compared genomic ancestry (*Materials and Methods*) to heterozygosity in sliding windows (size = 100 kb, step = 10 kb). We found that the regions that are homozygous for FL ancestry are almost completely depleted of heterozygosity, and reside in long ROHs (Fig. 3 *A* and *B*). Within PTFP individuals, the distribution of heterozygosity in windows with homozygous FL ancestry (Fig. 3*B*) looks similar to that of CFPs (Fig. 2*B*).

We did not find any regions in our genomic sequences (confidence inferences with posterior probability >95%) where the local ancestry inferences of PTFP individuals were completely replaced by Texas ancestry, which would be indicative of genetic swamping (Fig. 3*C*). This demonstrates that local alleles are being retained in the Florida population post-genetic-rescue.

### Simulated Effects of Genetic Rescue on Heterozygosity and Fitness Detected.

**Fig. 4.**
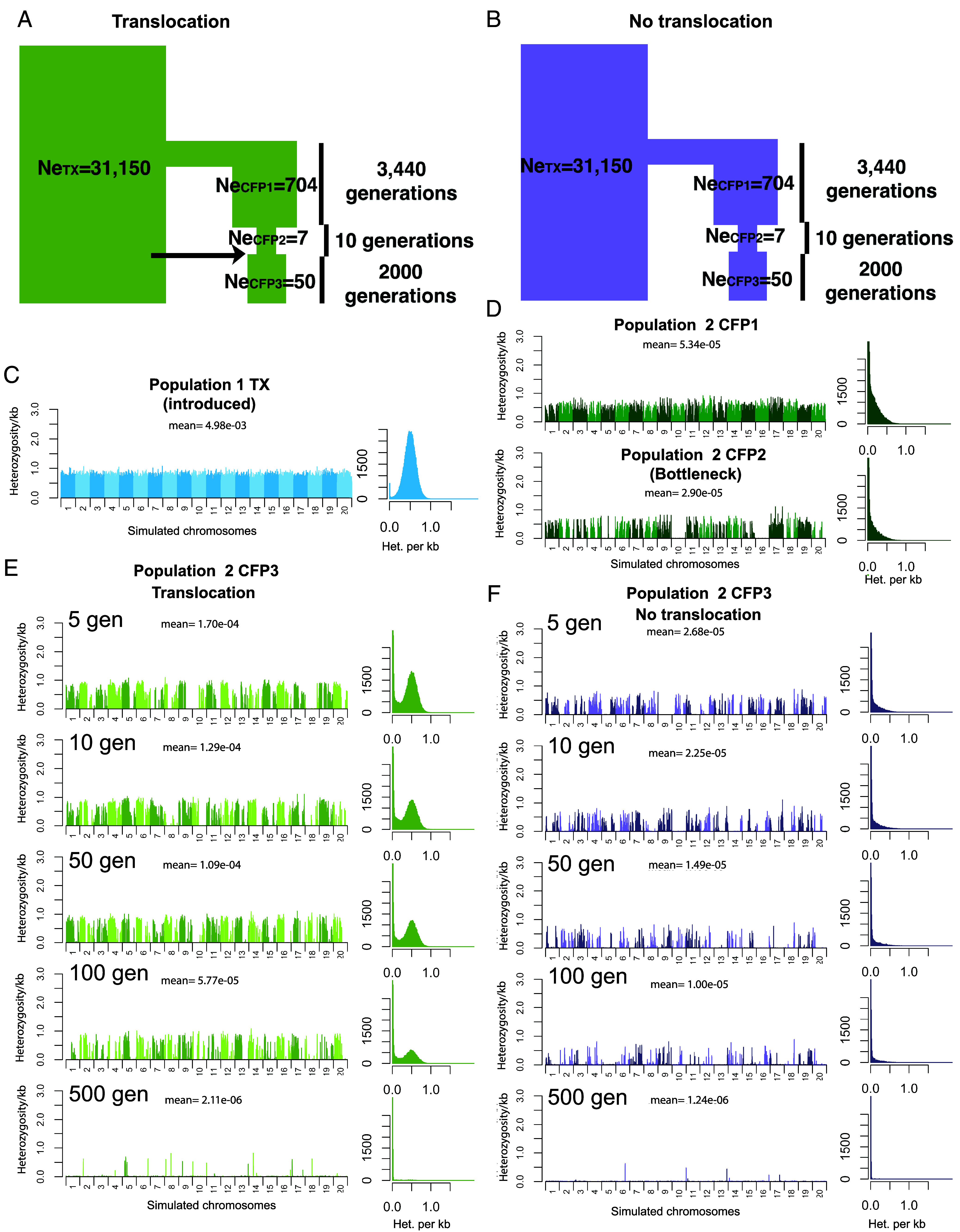
Simulations of ancestral demography and translocations and their effect on genome-wide heterozygosity under a partially recessive model. (*A*) Model of CFP-TX with translocation. (*B*) Model of CFP-TX with no translocation. Panels *C*–*F* show a Manhattan plot of the genome-wide heterozygosity and a histogram of those values to the right. Each panel shows one representative individual from each population and is labeled with the mean genome-wide heterozygosity. (*C*) Simulated TX population. (*D*) Simulated CFP population, *Top* panel before severe bottleneck (Ne_CFP1_ = 704), *Bottom* panel during severe bottleneck (Ne_CFP2_ = 7). (*E*) Simulated CFP population X number of generations after translocation and population expansion (Ne_CFP3_ = 50). (*F*) Simulated CFP population after expansion.

We used SLiM simulations (34) to predict how ancient demographic history and genetic rescue affects heterozygosity and fitness (35). We obtained effective population sizes (Ne) and divergence parameters from a previous study that used ∂a∂i to infer the demography taking into account inbreeding (36). We simulated the following scenarios: A) genetic rescue of CFPs by introduction of pumas from Texas (TX), and B) the same demographic model as A without any gene-flow (Fig. 4 *A* and *B*). In both scenarios, the simulated CFP population experienced a severe bottleneck (Ne_CFP2_ = 7) and then recovered to a very small population size (Ne_CFP3_ = 50). Scenario B serves as a baseline to evaluate how population size alone affects genetic variation and fitness, allowing us to assess the effects of the rescue. We simulated both scenarios under a partially recessive model dominance effect (*h* = 0.45, *s* > −0.001), (*h* = 0.2, −0.001 ≥ *s* > −0.01), (*h* = 0.05, −0.01 ≥ *s* > −0.1), (*h* = 0.0, *s* ≤ −0.1) (37) and a model with only additive effects (*h* = 0.5).

Considering heterozygosity, one generation prior to the severe bottleneck, heterozygosity is only slightly lower in the simulated CFP compared to the simulated TX population due to the different ancestral population sizes (Ne_TX_ = 31,150, Ne_CFP1_ = 704) (Fig. 4 *C* and *D*). The bottleneck has a similar impact on heterozygosity for both dominance models, (Fig. 4 and *SI Appendix*, Fig. S1*A*). At this time, the simulated CFP population has fairly uniform levels of heterozygosity across the genome with an average of 0.00005 per bp. During the bottleneck (Ne_CFP2_ = 7), heterozygosity decreases in certain segments of the genome, while remaining high in others (Fig. 4*D*). For both dominance models, the heterozygosity increases five generations after translocation by an order of magnitude (Fig. 4*E* and *SI Appendix*, Fig. S1*A*), to levels higher than those observed before the bottleneck. Further, gene flow reduced the number of segments of the genome with very low heterozygosity, resulting in a more pronounced bimodal distribution (Fig. 4*E*). Here, the patterns of heterozygosity in the simulated CFP genomes qualitatively mimic those of the empirical data (compare Fig. 4 *D* and *E* to Fig. 2*B*), suggesting that translocation is a plausible mechanism which led to the genome-wide patterns. Note that simulations without the translocation show low heterozygosity across the genome, and a unimodal distribution of heterozygosity (Fig. 4*F*), inconsistent with the empirical data (Fig. 2*B*). As time since the bottleneck increases, heterozygosity decays in both scenarios.

**Fig. 5.**
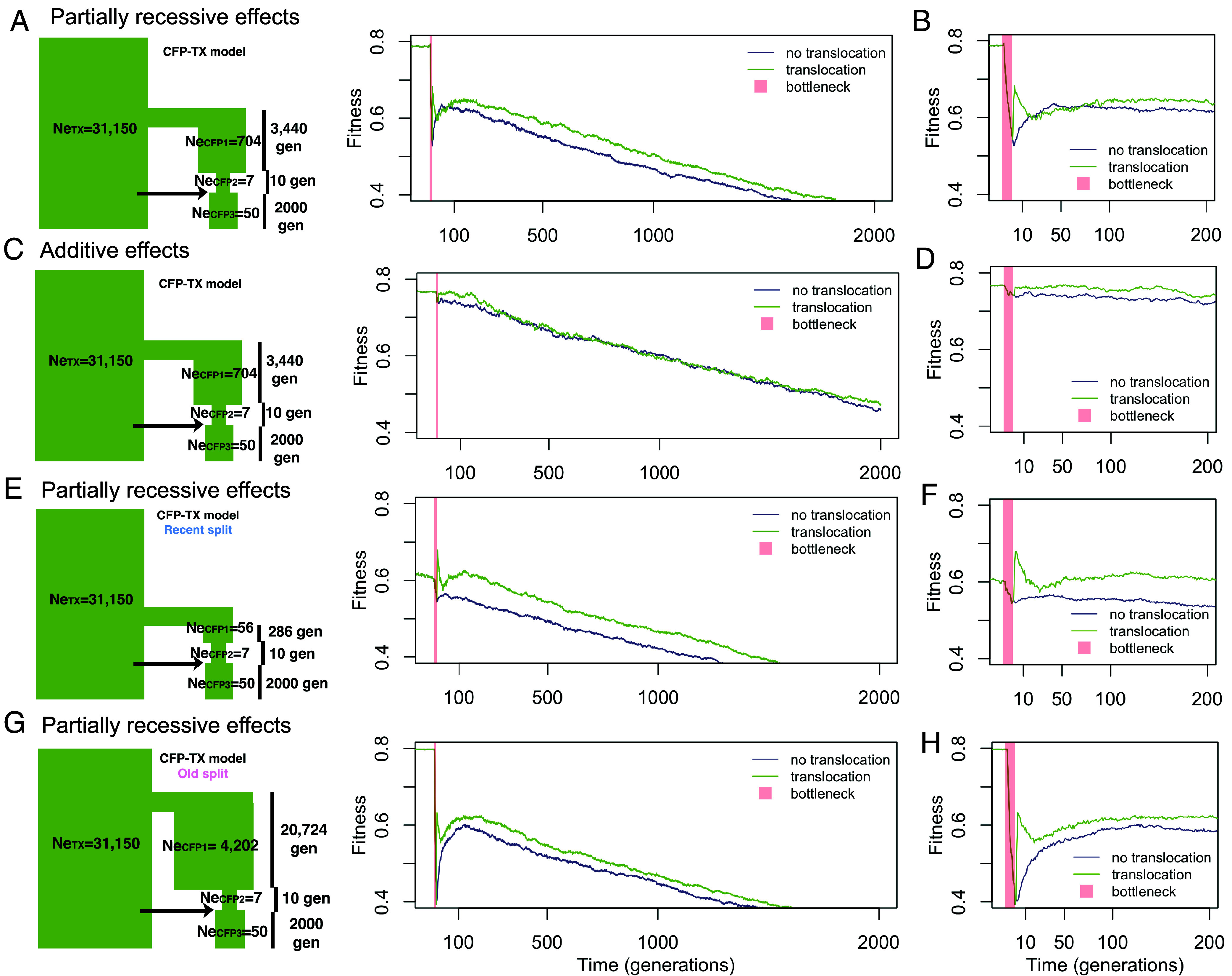
Average fitness over time in simulations. Fitness is scaled relative to an individual with no deleterious variants. Models with translocation are in green and models with no translocation in are purple. *Left* panels (*A*, *C*, *E*, and *G*) show the demographic model and a plot of the fitness starting before the severe bottleneck (Ne_CFP2_) until 2,000 generations after the population expansion (Ne_CFP3_), *Right* panels (*B*, *D*, *F*, and *H*) display the fitness in the first 200 generations after the bottleneck (Ne_CFP3_). (*A* and *B*) Partially recessive model of the original model. (*C* and *D*) Additive effects model in the same model. (*E* and *F*) Partially recessive model with more recent split time between TX and CFP. (*G* and *H*) Partially recessive model with older split time between TX and CFP.

Considering fitness, immediately after the bottleneck, there is a reduction in fitness in CFP in the model involving partially recessive effects (Fig. 5 *A* and *B*), but not in the model with only additive effects (Fig. 5 *C* and *D*) suggesting a higher load due to partially recessive deleterious variants being exposed in the homozygous state in CFP (*SI Appendix*, Table S3). On average, in all models, the translocation scenario resulted in genetic rescue (increased fitness compared to the no translocation scenario). However, this increase in fitness is transient, as fitness drops again within 20 generations of gene flow (Fig. 5 *A* and *B*). In the model without translocation, fitness increases more slowly after the bottleneck, and about 100 generations after the bottleneck, the fitness after translocation is only slightly higher than that without the translocation (Fig. 5 *A* and *B*). The increase in fitness is transient because the population size remains small (Ne_CFP3_ = 50), increasing homozygosity for deleterious variants over time. Indeed, the slight long-term increase in fitness due to translocation is not seen consistently across all individual simulation replicates, suggesting that the long-term benefits of translocation are subject to chance (*SI Appendix*, Fig. S4). In the additive model, fitness is more stable over time, although it slowly decreases due to accumulation of deleterious variants (Fig. 5 *C* and *D*). In sum, our results suggest that the observed benefits of genetic rescue are likely due to increased heterozygosity dampening the effects of recessive deleterious variants that otherwise would be exposed in the homozygous state.

To account for the parameter uncertainty, we also implemented models with different demographic scenarios: one with a more recent split between CFP and TX and smaller ancestral population size before the bottleneck (Fig. 5 *E* and *F*); another with an older split time and larger ancestral population time (Fig. 5 *G* and *H*). In terms of the patterns of heterozygosity, both models have similar results to the original model (*SI Appendix*, Fig. S1). When considering fitness, both scenarios have a sharp increase in fitness after translocation, followed by a decline, mimicking the original model. This pattern is likely due to the initial alleviation of homozygosity by introducing new alleles; the drop is a consequence of introducing all types of alleles, including deleterious ones. The recent split model had a lower fitness before the translocation (*SI Appendix*, Table S3 and Fig. 5*E*) due to its smaller population size (Ne_CFP1_ = 56). Interestingly, the difference in fitness between the translocation and no translocation scenarios is the largest in this model. Perhaps the population with the lower starting fitness can be better rescued by translocation. Alternatively, it might suggest that translocation from a less diverged population is more effective in genetic rescue.

### Deleterious variation in empirical puma genomes after genetic rescue.

We assessed the patterns of putatively deleterious amino acid changing variants across the puma genome. To do this, we annotated variants that fell in coding regions using SIFT (38) to predict whether they are deleterious or tolerated. We found that when comparing the ratio of deleterious/tolerated alleles within individuals, Texas (TX) had a lower proportion of deleterious alleles than CFPs, though this difference was slight and not statistically significant (Wilcoxon CFP-TX *P*-value = 0.1111, *SI Appendix*, Fig. S2*A*). The PTFPs had a slightly higher proportion of deleterious alleles than CFP, but this difference was also not significant (Wilcoxon CFP-PTFP *P*-value = 0.5649, *SI Appendix*, Fig. S2*A*). Taking into account only homozygous genotypes, TX had the lowest proportion of homozygous deleterious variation and the differences with the Florida populations were significant (Wilcoxon PTFP-TX: *P*-value = 0.00104 Wilcoxon CFP-TX: *P*-value = 0.01587, *SI Appendix*, Fig. S2*B*). After rescue, Florida panthers had a decreased proportion of homozygous deleterious variation compared to before genetic rescue (Wilcoxon CFP-PTFP *P*-value = 0.00069, *SI Appendix*, Fig. S2*B*). When we assessed the loss of function (LOF) variants annotated using snpEff (39, 40) there was an increase in the number of derived LOF alleles per individual after the rescue (Wilcoxon CFP-PTFP *P*-value = 0.005921, *SI Appendix*, Fig. S2*D*), but fewer homozygous LOF genotypes (Wilcoxon CFP-PTFP *P*-value = 0.01646, *SI Appendix*, Fig. S2*E*).

In PTFP individuals, we found a significant correlation between deleterious homozygous genotypes and the proportion of the genome with no TX ancestry (R^2^ = 0.65, *P*-value = 6.2e-08). We also found a significant correlation between homozygous LOF genotypes and the proportion of the genome with no TX ancestry (R^2^ = 0.41, *P*-value = 0.00016). The individuals with less TX ancestry have more homozygous deleterious and LOF variants (*SI Appendix*, Fig. S2). However, we observed similar correlations between homozygous Florida ancestry and predicted tolerated variants (R^2^ = 0.37, *P*-value = 0.00036, *SI Appendix*, Fig. S2 *J*–*L*), suggesting that the decrease in homozygosity is a genome-wide phenomena driven by admixture.

### Selection for Ancestry Postgenetic Rescue.

**Fig. 6.**
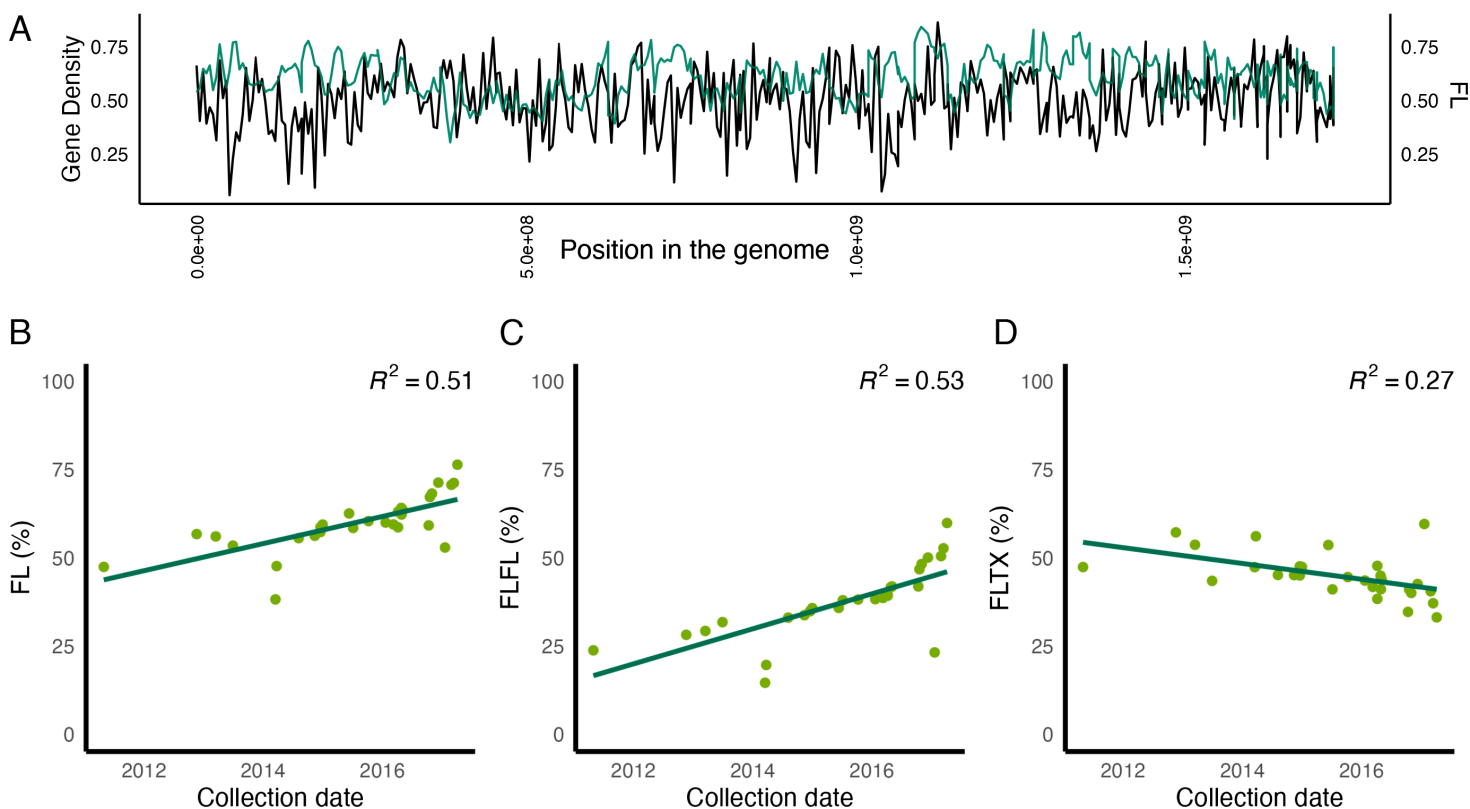
Testing for natural selection after genetic rescue. (*A*) Gene density (proportion of a 100 Kb window that is covered by a gene) in black and proportion of individuals with Florida ancestry in each window in green. (*B*) Collection date of PTFPs and Florida ancestry (FL%). (*C*) Collection date of PTFP and homozygous Florida ancestry (FLFL%). (*D*) Collection date of PTFP and heterozygous ancestry (FLTX%).

While we found no evidence of genetic swamping (Fig. 3*C*), we were interested in the possibility of selection after rescue leading to an enrichment of ancestry depending on the local gene density in the genome. An increase in Texas (TX) ancestry in gene-rich regions would indicate that selection might be acting to increase TX ancestry in functionally important regions. We divided the genome into nonoverlapping windows of 5 Mb, calculated gene density and ancestry proportion in each window (*Materials and Methods*), and investigated the correlation between these two variables (Fig. 6*A*). We found that gene density and ancestry are not significantly correlated at this scale (R^2^ = 0.00021, *P*-value = 0.7692). Thus, we do not find evidence that selection has favored replacement of original Florida DNA with Texas DNA in any systematic fashion. This further supports the hypothesis that the beneficial effect of genetic rescue is to increase heterozygosity, diminishing the effect of recessive deleterious alleles, rather than reducing the overall number of deleterious alleles.

To assess whether the proportions of Florida and Texas ancestry are shifting over time, we analyzed the correlation between collection date and ancestry proportions of PTFPs, excluding F1 individuals. Our results indicate an increase in Florida ancestry over time (Fig. 6). More recently collected panthers exhibit higher Florida ancestry (Fig. 6 *B* and *C*) and show fewer genomic regions with admixed ancestry (Fig. 6*D*). While this pattern could be explained by a number of factors (*Discussion*), we searched for signals of positive selection using several selection scan methods (*SI Appendix*, Fig. S3). However, we did not find any obvious signals of local selection.

## Discussion

Here, we combined whole-genome sequence data with population genetic simulations to investigate the genomic impact of genetic rescue in Florida panthers. Overall, we found that the translocation had several beneficial effects including a reduction in homozygous genotypes for deleterious variants, an increase in genetic variation, a reduction of relatedness, and a reduction of ROHs. The long-term (>50 generations into the future) benefits of the translocation on population fitness and heterozygosity are less certain, however.

One of the major concerns when translocating individuals for genetic rescue is the potential for genetic swamping, where the original ancestry is completely replaced by ancestry from the migrant population (22, 24, 25). The genetic rescue of Florida panthers was necessitated by predictions that the population would become extinct within a century without some form of management initiative (11). Translocation with the intent of genetic rescue was implemented by releasing wild female pumas from Texas into South Florida without knowing whether there were important adaptive differences between the two populations. Identifying adaptive variation specific to Florida panthers may be important to preserving local variation. Almost thirty years after the translocations in 1995, our genetic analysis of 31 PTFPs showed that these admixed individuals retain on average 59%-80% Florida ancestry (*SI Appendix*, Table S1). Moreover, we found that no region of the genome in all Florida panthers was completely replaced by Texas ancestry, indicating that local variation has been preserved with no evidence to date of genetic swamping. On the contrary, we found that Florida ancestry has increased over time following the implementation of genetic rescue (Fig. 6 *B* and *C*). A similar observation was made by Onorato et al. (16). One of the objectives of the original plan for genetic rescue was to achieve a level of 20% admixture (12). However, multiple studies have documented levels of admixture above 50% following translocation (13, 16), and we observe a strong subsequent decline in Texas ancestry (Fig. 6 *B*–*D*).

The decline in Texas ancestry could be driven by several factors. First, while Texas ancestry may have been initially favored in the Florida population to reduce homozygosity of recessive deleterious variants, it may have brought in novel recessive deleterious variants. Over time, homozygosity for Texas ancestry could have reduced fitness due to homozygosity for these deleterious variants. Importantly, the Texas population likely carried more recessive deleterious variants in the heterozygous state than the prerescue Florida population due to larger population size and higher heterozygosity in the former. Thus, homozygosity for Texas ancestry is predicted to result in more inbreeding depression than homozygosity for FL ancestry (41). Second, it is possible that there are locally adapted alleles in the original Florida ancestry that were not present in the translocated Texas pumas. Consequently, Texas ancestry would not have been favored in Florida and was selected against. Additionally, cryptic biases in the sampling of panthers over time could be driving this apparent change in ancestry proportions. A final explanation has to do with declining breeding success of the translocated Texas females in Florida. Only five of the eight Texas females were documented to have produced litters and there may have been a tendency of declining reproductive success of these females through time. Four of the Texas pumas died within 5 y of their release in 1995, reducing the probability of additional admixture. We note that a multitude of studies have documented the benefits of genetic rescue to this population (13, 14, 16, 42) regardless of the level of admixture and we urge caution in not overinterpreting these changes in ancestry over time. Continued study of the Florida panthers and sequencing of generations postgenetic rescue could help determine whether these proportions might change in the future.

We did not find evidence of systematic replacement of ancestry in coding regions. Our results indicate that the proportion of Florida or Texas ancestry is not correlated with gene density. Additionally, we did not find a significant difference in the number of deleterious alleles in Texas and Florida panthers prior to genetic rescue. Both populations had similar levels of deleterious genetic variation (*SI Appendix*, Fig. S3 *A*, *D*, and *G*), however, Florida panthers had substantially higher homozygosity. While these results seem to indicate no ongoing selection for ancestry genome-wide in coding regions, we cannot dismiss the possibility that there are particular regions of the genome where selection has favored Texas or Florida ancestry. However, since the translocation is so recent, detecting specific loci under selection is challenging due to linkage disequilibrium not being broken apart in such few generations (43).

We found no differences in the total number of deleterious alleles per individual in Florida panthers before and after genetic rescue. Instead, a reduction in homozygosity seems to be driving improvements in fitness by reducing the recessive deleterious load. Our results show that PTFPs have much higher heterozygosity and fewer ROHs than CFPs. Our simulations also support these findings, predicting a marked impact of genetic rescue in the presence of recessive variants, but very little impact when only additive variants are present. Finally, simulations predicted that while the genetic rescue was extremely effective at increasing heterozygosity and reducing ROHs, fitness will continue to decrease over the long-term if the population remains small and isolated from conspecifics. This implies that the population will require continued management to remain viable, a supposition that has also been identified in recent population viability models of Florida panthers (14).

Our simulations show that even one single pulse of gene flow (translocations) can have long lasting effects on the heterozygosity (100 generations) (Fig. 4*E* and *SI Appendix*, Fig. S1). However, this finding is sensitive to many parameters, including *N_e_* and reproductive success, and is probably an optimistic scenario. After 500 generations, in all simulation scenarios, a population of 50 individuals will inevitably suffer an almost complete loss of heterozygosity across the genome and low fitness. These results indicate that a larger population size would be required to maintain heterozygosity and high fitness of the population for extended periods of time. Alternatively, additional translocations for the purpose of genetic rescue could be performed to increase these quantities, although such an approach also has the potential to introduce new deleterious variants. Further analyses are needed to investigate how future genetic rescue attempts would benefit the population.

Our findings agree with previous research on the positive effects of translocation in Florida panthers, resulting in genetic rescue (7, 13–16). However, our genetic load analysis and simulations find that many of these effects might be transient, only temporarily increasing fitness, and that continued management of the Florida panther will be key to population persistence and recovery. Our simulations show that the translocation on average is beneficial under a wide range of demographic assumptions. However, the long-term benefits of translocations (i.e., >50 generations) on fitness are highly stochastic (*SI Appendix*, Fig. S4). Furthermore, even though translocation is beneficial under the scenarios we have studied, in some cases, such a management initiative may not lead to increased fitness as the specific effects depend on the random distribution of deleterious variants in the genome. These results highlight the inherent uncertainty in the long-term success of translocations. In other species and populations of conservation concern where translocations are being considered, we recommend studies of the deleterious load in both donor and recipient populations before genetic rescue is initiated.

## Materials and Methods

### Collection of Samples.

Field methods used to capture Florida panthers and collect biomedical samples—including blood, tissue and hair for DNA samples—are described in van de Kerk et al. (14). Most of the PTFP samples in this study (26/31) were uncollared individual mortalities and are labeled as (UCFP). A majority of the UCFP samples were roadkills reported by the general public (see Dataset S1 for more sample details).

### DNA, Library Preparation, and Sequencing.

We extracted high molecular weight DNA from 29 *P. concolor* individuals. We then sonicated the purified DNA using a Q800R3 Sonicator (QSonica). We sonicated DNA samples for nine minutes total ON time, with a pulse of 15 s ON/15 s OFF, 40% amplitude. Tubes were spun down in a centrifuge every three minutes and then restarted. The target fragment size was ~300 to 500 bp, and we verified this by running 1 μL in a ~1% agarose gel. Sonication was followed by double-sized size selection using lab-made SPRI beads [20% PEG-8000/2.5 M NaCl/1 mg/mL Sera-Mag SpeedBead Carboxylate-Modified Magnetic Particles (Hydrophobic) 65152105050250 (44)] to refine the fragment sizing. These magnetic beads reversibly bind to larger or smaller-sized DNA fragments depending on the volume added. The target size for our libraries was ~350 bp, so we used a 0.5× ratio for the right-side selection and 0.65× for the left-side. We used the Rainin Benchsmart 96, a high-throughput pipetting system to treat samples simultaneously. Then, we used a Kapa HyperPrep library prep kit (Roche Co.) for end repair and A-tailing, followed by adapter ligation with a universal stub. The next step was a postligation bead clean-up to remove excess adapters and ligase. Finally, we performed a 0.8× SPRI cleaning on the Benchsmart.

We performed an indexing PCR with eight cycles. We used a plate provided by the Berkeley Genome Sequencing Facility that contained a premixed unique P5 and P7 indexing oligo for each sample. The Benchsmart was again used to perform a final 0.8× bead clean-up to remove excess indexing oligos and dimers. The target size of fragments after adapter and index is ~450 to 650 bp. After completing the libraries, we performed quality control by measuring the final concentration using a plate reader and Bioanalyzer DNA 1,000 chips to obtain library sizing information. The libraries ranged between 417 and 480 bp, with an adapter of 135 bp, meaning our inserts were ~280 to 345 bp. We sequenced all libraries on one lane of Illumina NovaSeq 6000 150PE Flow Cell S4.

### Read Cleaning and Mapping.

We used Trimmomatic-0.39 to remove adapters (ILLUMINACLIP:TruSeq3-PE.fa:2:30:10), trim leading and trailing low quality or N bases (below quality 3) (LEADING:3, TRAILING:3) or when the average quality per base drops below 15 in a 4-base sliding window (SLIDINGWINDOW:4:15), and drop reads <75 bp in length (MINLEN:75). All reads were mapped to the *P. yagouaroundi* reference genome GCF_014898765.1 (45)(NCBI Accession No. PRJNA717316), which is an outgroup, using bwa mem. We intentionally used an outgroup as the reference to ensure all populations are equally diverged from the reference. This also allows us to interpret the alleles that match to the reference as ancestral and the nonreference as derived. The mapped alignment files were viewed with the command “samtools view -Sb -F 1804,” followed by sorting and indexing. The binary filter flag -F 1804 excludes unmapped reads (0 × 4), mate unmapped (0 × 8), not primary alignment (0 × 100), reads that fail platform/vendor quality checks (0 × 200) and reads that are PCR or optical duplicate (0 × 400). We applied these methods to the data that we generated as well as to the publicly available reads we downloaded from Saremi et al. (8) and Ochoa et al. (7). We analyzed 50 individuals in total (Dataset S1), 29 are unique to this study.

### Variant Calling and SNP Filtering.

We used GATK 4.2 (46) HaplotypeCaller to produce gVCF files for the 50 individuals using the options *-*ERC BP_RESOLUTION, minimum mapping quality set to 30 and minimum base quality score set to 25. We then generated joint VCF files by passing all individual gVCF files to GATK GenotypeGVCFs using the options -allSites and -stand_call_conf *0*. We left aligned and trimmed variants (LeftAlignTrim). We then used a custom filtering script to keep invariant sites and biallelic SNPs. We excluded genotypes with insufficient or excess read depth (<1/3× or >2× mean read depth for that individual). We excluded sites that did not pass our filtering criteria (QD < 4, FS > 60, MQ < 40, MQRankSum < −12.5, ReadPosRankSum < −8, SOR > 3), with excess missingness (>25%) or excess heterozygosity (>75%). We excluded repeat regions masked by NCBI (identified by Window Masker and additionally masked repetitive sequences identified with RepeatMasker.

Scaffolds that were putatively derived from sex chromosomes or mitochondria were excluded using Plink (v1.90b6.26) (47). For sex chromosomes, we picked 5 PTFP males and 5 PTFP females and plotted the normalized read coverage along the scaffolds using windows (bedtools makewindows -w 100000 -s 10000). When 80% of the windows on a scaffold have an “abnormal” ratio (i.e., male/female coverage >1.15 or <0.85), the scaffold was defined as sex-chromosome-related scaffold. All SNPs from the sex-related scaffolds or windows with abnormal coverage ratio were removed. For mitochondria, scaffold NC_028311.1 was excluded.

### Variant Annotation.

We used the *P. yagouaroundi* genome and its RefSeq annotation to predict the effects of variants and annotate our vcf files. We used SIFT, a homology-based method, to determine whether an amino acid substitution is deleterious or tolerated (38). To identify LOF variants we used SnpEff (39, 40). We normalized counts (C) within each individual dividing by the total number of sites that pass all the filters in each individual (i). This helps account for different numbers of variants due to differences in coverage. We scaled all of the normalized counts by multiplying by the mean number of sites that pass all the filters (μ_i_). The derived allele was defined as the allele that does not match the jaguarundi reference genome (derived = 1, ancestral = 0), as the reference is equidistant to all ingroup puma samples. To count total number of derived alleles (*a*) per individual in each category we used the formula:

$$a=\left(\frac{C_{0/1}}{i}+2\left(\frac{C_{1/1}}{i}\right)\right)*\mu_{i},$$

where $C_{0/1}$ is the count of heterozygous genotypes and $C_{1/1}$ is the count of homozygous derived genotypes. To count derived homozygous genotypes (*hom*) we used the following formula:

$$hom=\left(\frac{C_{1/1}}{i}\right)*\mu_{i}.$$

### Population Structure.

We used plink (v1.90b6.26) to apply a minor allele frequency filter (--maf 0.05). Additionally, all variants with one or more multicharacter allele codes (--snps-only) or all variants where at least two alternative alleles are present in the dataset (--biallelic-only) were excluded. All variants with missing call rates exceeding 0.5 were excluded (--geno 0.5). We generated the PCA using these sites using plink’s pca command (--pca).

We used angsd (48) to generate a beagle file with the same sites and generate genotype likelihoods. The beagle file was converted to lgm format using ped2lgm before feeding into qpas, ancestry component command from OHANA (31). Qpas was run with k = 3, 4. The ancestry component plot was generated using pong (49).

### Local Ancestry inference.

The chromosome painting was performed using ancestryHMM (33) software and genotype calls. To prepare the data for chromosome painting, the filtered sites were pruned to remove variants in linkage disequilibrium (LD). Variants were removed using plink --indep-pairwise “10 kb 1 .16,” by setting the window size to be 10 kb, the step size to be 1 variant count, and R^2^ threshold to 0.16. Using the python script vcf2ahmm.py (https://github.com/russcd/Ancestry_HMM/scripts), the sites were further filtered by setting the minimum number of samples in each ancestral population to consider a site to 4 (--min_total 4) and the minimum allele frequency difference between any pair of ancestral populations to include a site to 0.2 (--min_diff 0.2). A total of 284,228 variants passed all filters and were used as input for ancestryHMM. We ran ancestryHMM with the following setting: the overall ancestry proportion of PTFPs was set to be 80% Florida as the indigenous ancestry and 20% Texas, and the admixture time was set five generations ago. These parameter settings were inspired by the OHANA results. The error rate per site was estimated to be 0.01. For each individual, we only kept the sites that had one of the states (0/0,0/1,1/1), with a posterior probability >95%. We grouped adjacent SNPs with same ancestry to create intervals; all the positions between the two adjacent SNPs (with no call of ancestry) were assumed to share the same ancestry as their flanking SNPs. These intervals of ancestry were used to compare heterozygosity and ancestry.

### Heterozygosity, ROHs, and Relatedness.

We calculated the heterozygosity as the proportion of all called genotypes within a single individual that are heterozygous (different between each pair of homologous chromosomes). We used sliding windows across the genome (size = 100 kb, step = 10 kb). Within each window, we calculated the heterozygosity by dividing the count of heterozygotes sites by the total number of called sites.

We calculated relatedness using ngsRelate (32), which uses genotype likelihoods and accounts for inbreeding within a population. We used option -e 0.01 and a minor allele frequency filter of 5%. We identified ROHs with Bcftools (50) roh with option -G 30. To calculate the fraction of the genome in ROH (FROH), we divided the total number of base pairs in ROHs larger than 1 Mb, by the total length of the genome.

### Simulations.

We simulated the following scenarios: A) Introduction of pumas from TX (translocation) to Florida, and B) the same demographic model as A without translocation. We scaled the parameters by multiplying all population sizes and divergence times by a factor *m* from the ∂a∂i model (36) inferred for CFP and TX. Here, *m* is the ratio of the mutation rate used in that study and the mutation rate (0.5 × 10^−8^) that we use in this study based on Saremi et al. (8). For simulating the translocation, we used one single pulse of admixture with exactly five individuals from TX based on the number of Texas pumas that produced offspring (12, 13). For the population sizes of Florida during the severe bottleneck (N_FL2_ = 7) and after the population expansion (N_FL3_ = 50), we used estimates of the FL census (13) divided by four (51). We considered additional scenarios to account for the uncertainty in the ancestral parameters (*SI Appendix*, Table S2). Wright–Fisher simulations were conducted using SLiM v4.0.1 (34). We assumed a mutation rate of 0.5 × 10^−8^ per site per generation and a constant recombination rate of 1 × 10^−8^ per base pair per generation. We simulated 50 Mbp in each replicate, assuming 10%/90% coding regions and noncoding regions, respectively. We assumed that all mutations accumulating in coding regions were deleterious with a gamma-distributed distribution of fitness effect (DFE) with mean −0.043 and shape parameter α = 0.23, estimated by Eyre-Walker et al. (52). We evaluated two dominance effects models: one assuming additive deleterious mutations (*h* = 0.5), and one assuming partially recessive dominance effects determined by the severity of deleterious mutations, where *h* determined by *s* [(*h* = 0.45, *s* > −0.001), (*h* = 0.2, −0.001 ≥ *s* > −0.01), (*h* = 0.05, −0.01 ≥ *s* > −0.1), (*h* = 0.0, *s* ≤ −0.1) (37)]. Mutations accumulating on noncoding regions were assumed to be neutral (*s* = 0). We assumed log additive interactions among loci under selection, individual fitness (*f*) in simulations could be calculated by

$$f=\prod_{i\;\in \;heterozygous\;sites}\left(1+h_{i}s_{i}\right)\prod_{j\;\in \;homozygous\;sites}\left(1+s_{j}\right).$$

At the beginning of the simulation, all individuals have fitness value 1.0. As variants accumulate over time, the individual fitness changes based on the variants in the genome and their selection coefficients (*s*) and dominance coefficients (*h*). Recalculation of individual fitness for each individual is done in each generation of the simulation with the formula provided above, and an average of individual fitness from the donor and recipient populations are recorded throughout simulations. As each simulation contained a 50 Mb of the genome, we aggregated five replicates together by summing the log of the fitnesses across replicates. This resulted in the simulation of ~25 Mb coding region, approximately the size of a mammalian exome. We then generated 20 replicates for each scenario (*SI Appendix*, Fig. S4) and averaged these together for the results shown in Fig. 5.

To illustrate how genetic rescue affects the heterozygosity of the population, for each model, we randomly selected 20 diploid sampled individuals and output them in a vcf. We calculated the heterozygosity in 100 Kb windows, step 10 Kb using the same custom scripts that were used to calculate heterozygosity for the real data. We concatenated these 20 simulations spatially for each to represent a 50 Mb chromosome.

### Gene Density.

We used the RefSeq annotation of the *P. yagouaroundi* (NCBI Accession No. PRJNA717316). We extracted all the gene coordinates, by using a command line where the third column of the gff equaled “gene.” We used bedtools (53) to generate nonoverlapping windows of 5 Mb along the genome (-makewindows). We kept 5 Mb windows, discarding the fragments at the end of each scaffold that did not complete window length. Then, we used bedtools *coverage* to count the proportion of each window covered by gene annotations. Finally, we used bedtools intersect to include all of the SNPs analyzed with ancestryHMM that fell within each 5 Mb window. The ancestry of each SNP was calculated by counting all of the alleles of PTFP individuals from Florida and dividing by the total number of alleles with an ancestry assigned at each site. Ancestry was only assigned when the posterior probability was higher than 95%. Using *dplyr* library (54) and custom scripts in R, we averaged their ancestry to obtain a mean value of ancestry proportion per window. We compared the gene density and the ancestry proportion, and fitted a linear regression to test for a correlation.

### Selection Scans.

We used *angsd* (49) to perform selection scans using genotype likelihoods. First, we calculated the SFS and the 2d-SFS using *angsd -doSaf*, followed by *realSFS*. We performed a scan of F_ST_ in 100 Kb windows with 20 Kb steps (realSFS fst stats2 pop1.pop2.fst.idx -win 100000 -step 20000 -type 2) with the following combinations 1) CFPs − TX, 2) PTFPs − TX, and 3) PTFPs − CFPs. We used thetaStat do_stat to calculate Tajima’s D and average number of pairwise differences (tP) within each population (TX, CFP, and PTFP) in 100 Kb windows with 20 Kb steps.

## Disclaimers

Any use of trade, firm, or product names is for descriptive purposes only and does not imply endorsement by the U.S. Government.

Although this code has been processed successfully on a computer system at the University of California, no warranty expressed or implied is made regarding the display or utility of the data for other purposes, nor on all computer systems, nor shall the act of distribution constitute any such warranty. The USGS or the U.S. Government shall not be held liable for improper or incorrect use of the data described and/or contained herein.

## Supplementary Material

Appendix 01 (PDF)

Dataset S01 (XLSX)

Dataset S02 (XLSX)

## Data Availability

Reads and code data have been deposited in SRA (PRJNA898112) (29) and GitHub (https://github.com/aguilar-gomez/pumaRescue) (35), respectively.
