# Supplementary material for: Genetic rescue of Florida panthers reduced homozygosity but did not swamp ancestral genotypes
Source: Proc Natl Acad Sci U S A. 2025 Jul 28;122(31):e2410945122. doi: 10.1073/pnas.2410945122 (PMC12337334; doi:10.1073/pnas.2410945122)
Supplement: Supplementary file 1 — Appendix 01 (PDF) [file pnas.2410945122.sapp.pdf]

Supplementary Information for
Genetic rescue of Florida panthers reduced homozygosity but did not swamp ancestral genotypes

Diana Aguilar-Gómez[a,b,*], ID, Lin Yuan[c] ID, Yulin Zhang[b], Alexander Ochoa[d], Melanie Culver[e,f], Robert R. Fitak[g] ID, Dave Onorato[h] ID, Kirk E. Lohmueller[a,*] ID, and Rasmus Nielsen[b,*]

[a]Department of Ecology and Evolutionary Biology, University of California, Los Angeles, CA 90095
[b]Center for Computational Biology, College of Computing, Data Science and Society, University of California, Berkeley, CA 94720
[c]Cell and Molecular Biology Programme, School of Life Sciences, The Chinese University of Hong Kong, New Territories, Hong Kong Special Administrative Region of China
[d]Department of Ecology & Evolutionary Biology, Yale University, New Haven, CT 06520
[e]U.S. Geological Survey, Arizona Cooperative Fish and Wildlife Research Unit, University of Arizona, Tucson, AZ 85721
[f]School of Natural Resources and the Environment, University of Arizona, Tucson, AZ 85721
[g]Department of Biology, Genomics and Bioinformatics Cluster, University of Central Florida, Orlando, FL 32816
[h]Fish and Wildlife Research Institute, Florida Fish and Wildlife Conservation Commission, Naples, FL 34114

*corresponding authors: Diana Aguilar-Gómez, Kirk Lohmueller and Rasmus Nielsen

Email: aguilargomez@ucla.edu, rasmus_nielsen@berkeley.edu, klohmueller@ucla.edu

Author Contributions: R.N., K.E.L. and D.A.G designed the research. D.O. provided the samples. D.A.G did the library prep. D.A.G and L.Y. did all the bioinformatic analyses. Y.Z. performed the simulations. D.A.G, R.N., K.E.L., D.O. and A.O. wrote the paper. All authors read, gave feedback and approved the current version of the manuscript.

Competing Interest Statement: No competing interest.

Classification: Biological Sciences, Evolution, Genetics

Keywords: genetic rescue, puma, conservation genomics, genetic swamping

This PDF file includes:

- Supplementary Text
- Supplementary Figures 1 to 4
- Supplementary Tables 1 to 3

Supplementary Text

Ancestry discrepancies

In this study, we combined whole-genome sequence data with population genetic simulations to investigate the genomic impact of genetic rescue in the FL panthers. Some of the PTFP individuals do not appear admixed according to their genome-wide ancestry components as inferred using OHANA (Fig. 1D). In a recent study, it was similarly found that not all pumas post-genetic rescue had TX ancestry (1). However, AncestryHMM estimates all PTFPs to have Texas ancestry. In the two F_1 individuals, OHANA underestimates Texas ancestry to 40% and 44%, respectively, instead of the expected 50%, suggesting that the discrepancy is due to a downward bias in the OHANA analyses. OHANA is an unsupervised method and not even all TX individuals are inferred to have 100% TX ancestry (Fig. 1B), suggesting that the bias might be due to latent shared genetic variation between PTFP and Texas, possibly through gene-flow with a, now extinct, third population. The supervised analysis in ancestryHMM infers the genomes of both F_1s to be more than 91% of the diploid genomic positions FL/TX with 50% contribution from each ancestry. It underestimates the heterozygous sites (FL/TX) but correctly estimates 50% contribution from each parental population. For the local ancestry analysis, having a Central American sample as a third reference panel could result in more accurate estimates and possibly less discrepancies between OHANA and ancestryHMM. However, our results using South American pumas showed that PTFPs do not have a large proportion of this third ancestry.

The Florida Panthers at Everglades

Panthers historically present in Everglades National Park, including those with EVG ancestry (mixed canonical FP and Central American puma), face unique challenges, particularly due to their isolation from the core population of panthers (CFP and PTFP) in southwestern Florida resulting from the semi-permeable barrier associated with fluctuating water levels in Shark River Slough (Figure 1B) (2). Additionally, there is only a small portion of Everglades National Park that is considered quality habitat for panthers as most of Florida's east coast is heavily developed, severely limiting the local carrying capacity. The combination of limited gene flow and small population size in Everglades National Park (2, 3), likely has resulted in high levels of inbreeding in the area and, therefore, a high content of ROHs, as seen in our results and previous studies (4). Our findings of high heterozygosity and a high proportion of long ROHs in EVG can be reconciled by the demographic history including admixture in this region. They also match our simulation predictions, where it is possible to have higher heterozygosity and still present ROHs and lower fitness, although an even more extreme scenario is observed in EVG as it never experienced a population expansion after the bottleneck. Due to these factors, this subpopulation is likely to always be tenuous and even ephemeral (2) even with population management. Florida panthers in Big Cypress (CFP and PTFP) are likely less impacted because they are the main portion of the Florida panther population.

References

1. L. M. Penfold, *et al.*, Long-term evaluation of male Florida panther (Puma concolor coryi) reproductive parameters following genetic introgression. *J. Mammal.* **103**, 835–844 (2022).
2. D. Onorato, *et al.*, Long-term research on the Florida panther (Puma concolor coryi): historical findings and future obstacles to population persistence. *Biology and conservation of wild felids* 453–469 (2010).
3. D. P. Onorato, *et al.*, Habitat selection by critically endangered Florida panthers across the diel period: implications for land management and conservation. *Anim. Conserv.* **14**, 196–205 (2011).
4. N. F. Saremi, *et al.*, Puma genomes from North and South America provide insights into the genomic consequences of inbreeding. *Nat. Commun.* **10**, 4769 (2019).
5. P. D. Blischak, M. S. Barker, R. N. Gutenkunst, Inferring the Demographic History of Inbred Species from Genome-Wide SNP Frequency Data. *Mol. Biol. Evol.* **37**, 2124–2136 (2020).

6. A. Ochoa, D. P. Onorato, R. R. Fitak, M. E. Roelke-Parker, M. Culver, De Novo Assembly and Annotation from Parental and F1 Puma Genomes of the Florida Panther Genetic Restoration Program. *G3* **9**, 3531–3536 (2019).

Supplementary Figures and Tables

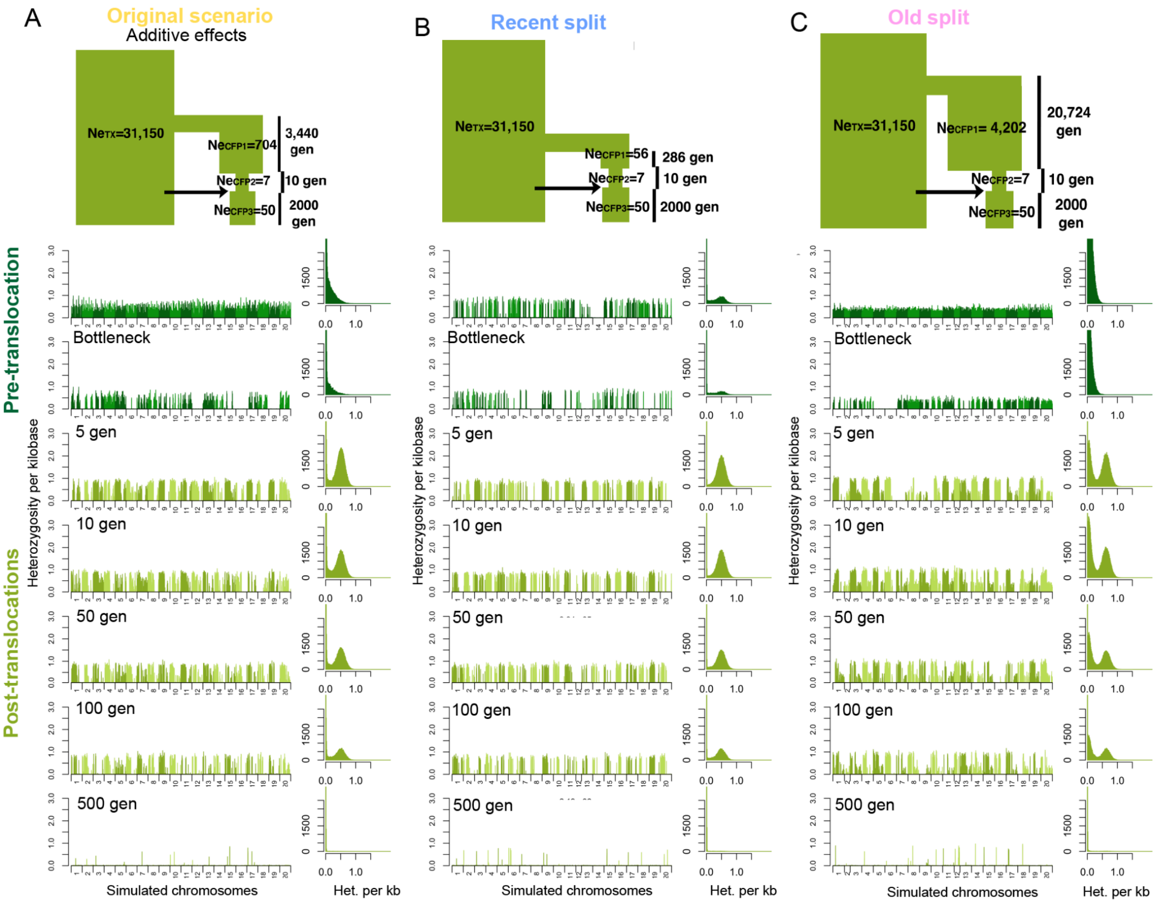

Supplemental Figure 1. Heterozygosity in additional models. A) Original model with additive dominance effects B) Model with a more recent split time between Texas and Florida populations. C) Model with older split time between Texas and Florida populations. See Supplementary Table 4 for the specific parameter values assumed.

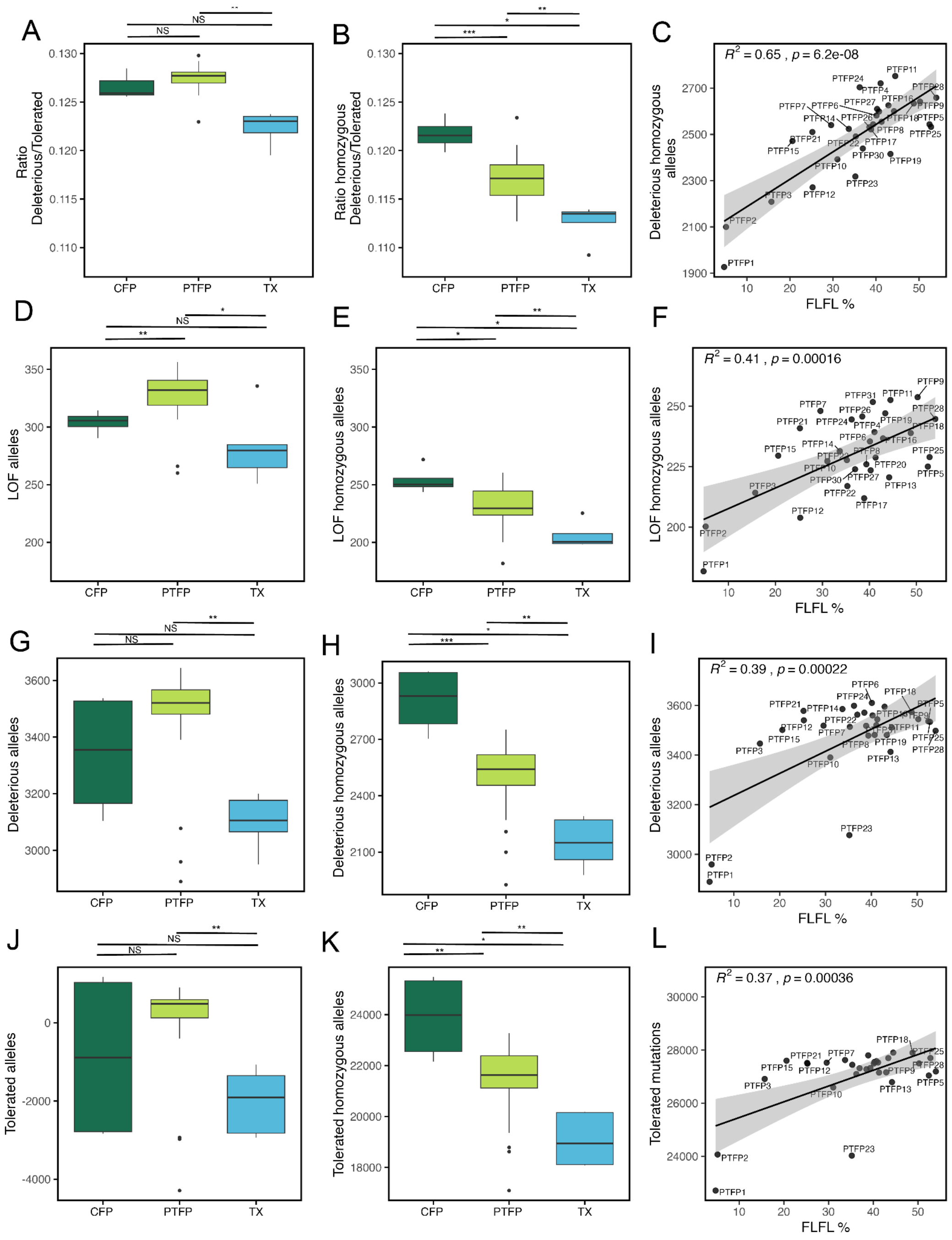

Supplementary Figure 2. Deleterious genetic variation. Boxplots with individuals separated by population Canonical Florida Panther (CFP), Post-translocation Florida Panther (PTFP) and Texas (TX). On top of each boxplot bars with Wilcoxon p-values: * <0.05, ** <0.01, *** <0.001, NS non-significant. A) Ratio of deleterious/tolerated all alleles B) Ratio of deleterious/tolerated homozygous alleles C) Linear regression, using only PTFP individuals: homozygous deleterious alleles ~ proportion of genome with homozygous ancestry from Florida (FLFL%) D) Loss of function (LOF) all alleles, E) LOF homozygous alleles, F) Linear regression, PTFP individuals: LOF homozygous alleles ~ FLFL% G) Deleterious alleles H) Deleterious homozygous allele I) Linear regression, PTFP individuals: deleterious alleles ~ FLFL% J) Tolerated alleles K) Tolerated homozygous allele L) Linear regression, PTFP individuals: tolerated alleles ~ FLFL%.

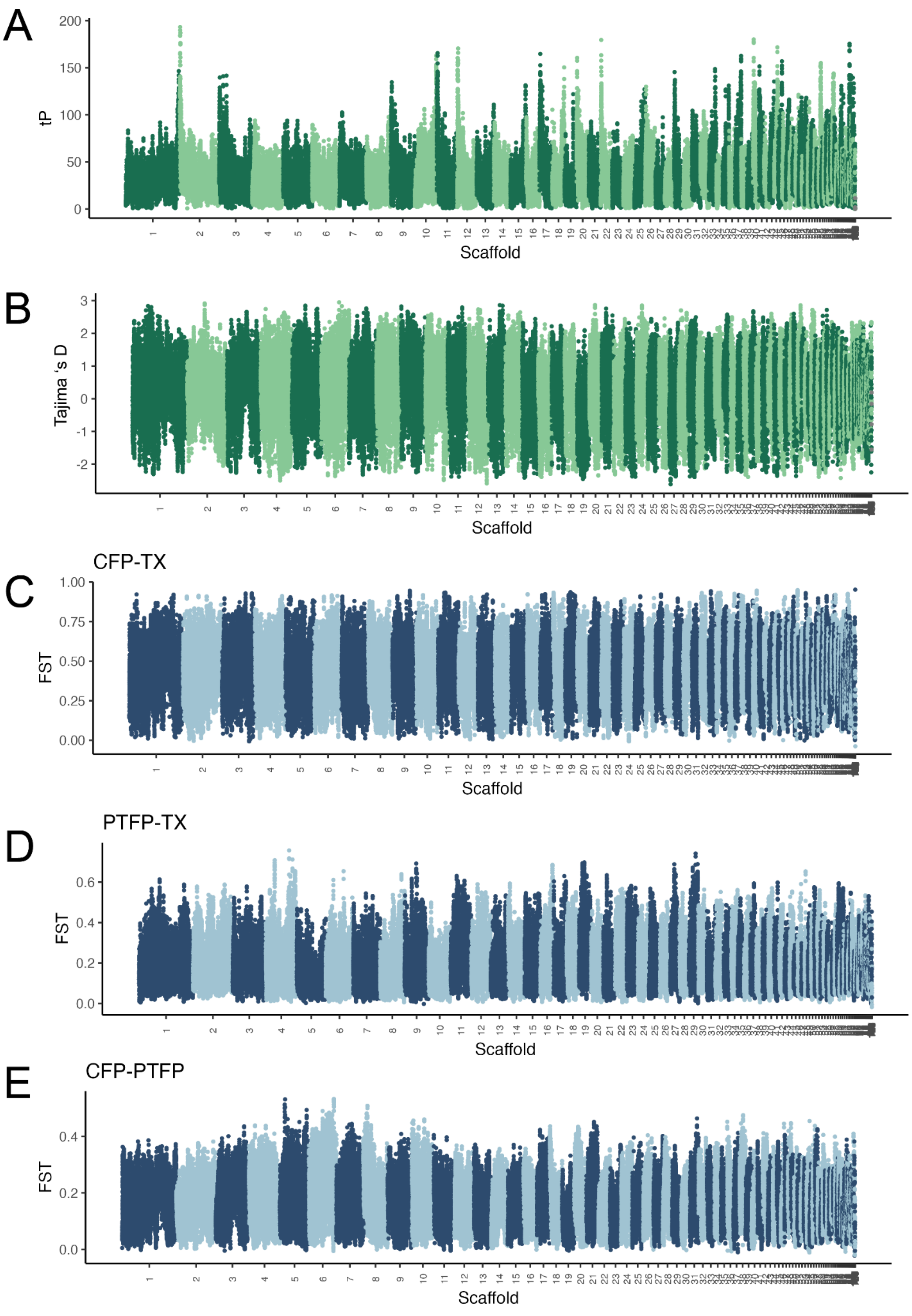

Supplementary Figure 3. Selection scans using 100Kb windows with 20kb steps. A) Average pairwise number of mutations (tP) in PTFP B) Tajima's D in PTFP C) F_{ST} scan between CFP and TX D) F_{ST} scan between PTFP and TX E) F_{ST} scan between CFP and PTFP.

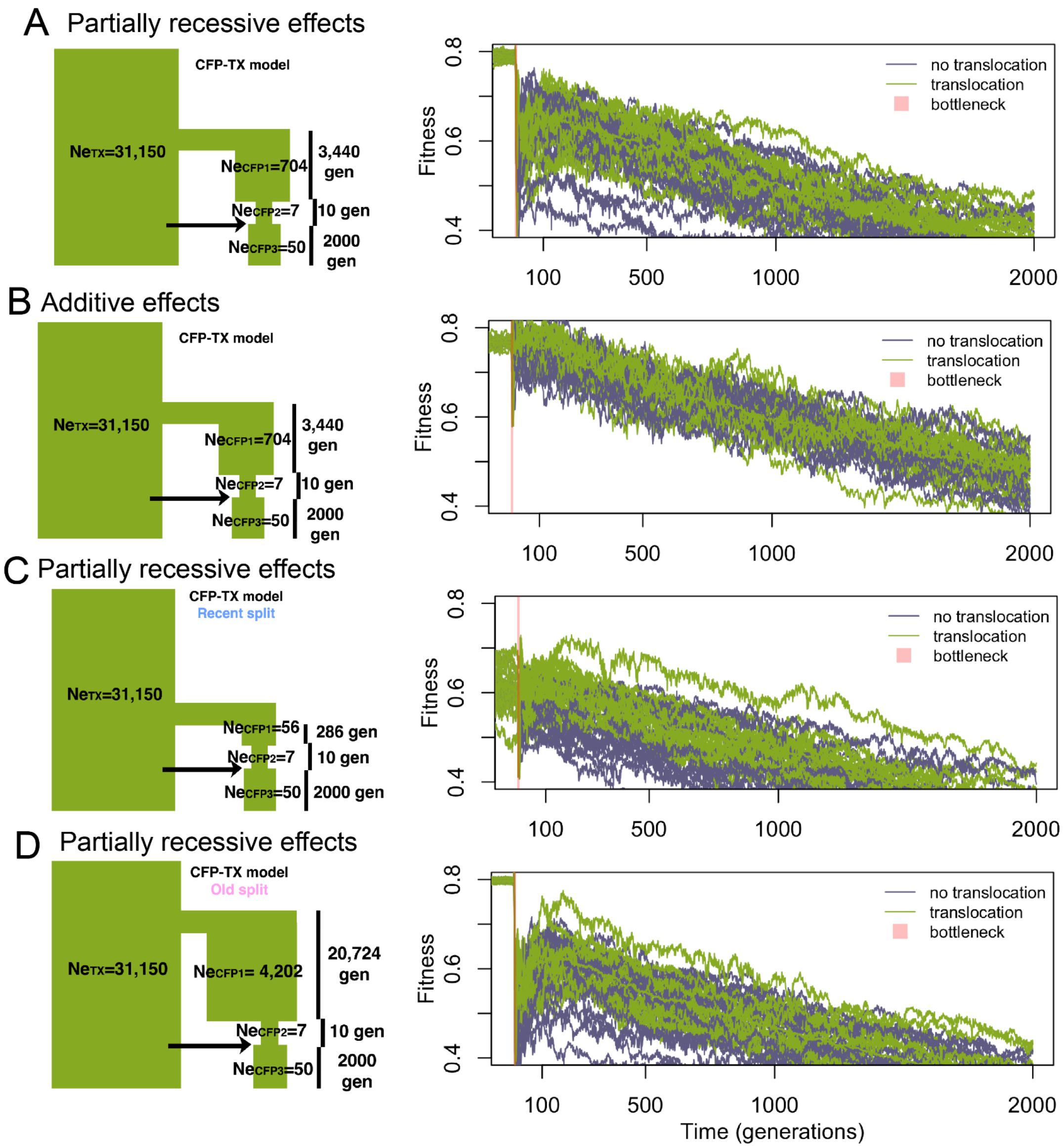

Supplementary Figure 4. Replicates of simulations. Demographic models used are shown on the left plots. Individual simulation replicates (each consisting of ~25Mb of coding sequence) are shown in the plots on the right. A) Partially recessive original model B) Additive effects original model C) Partially recessive recent split model D) Partially recessive old split model.

Supplementary Table 1. Inference of genome-wide ancestry using AncestryHMM and OHANA

	AncestryHMM					OHANA, k=4		
**Individual**	**FL**	**TX**	**FLFL**	**FLTX**	**TXTX**	**Florida**	**Texas**	**Collection date**
PTFP1	50	50	4	91	5	56	44	
PTFP10	56	44	29	54	17	64	36	3/16/2013
PTFP11	59	41	42	35	24	92	8	10/11/2016
PTFP12	53	47	23	60	17	79	9	1/19/2017
PTFP13	64	36	42	44	14	86	14	5/3/2016
PTFP14	53	47	32	43	25	94	6	6/29/2013
PTFP15	48	52	20	56	24	46	54	3/24/2014
PTFP16	64	36	42	45	13	81	19	4/26/2016
PTFP17	63	37	36	54	11	86	14	6/11/2015
PTFP18	67	33	47	41	12	99	1	10/18/2016
PTFP19	62	38	42	41	17	81	15	4/28/2016
PTFP2	50	50	4	92	4	60	40	
PTFP20	59	41	39	38	22	97	3	4/6/2016
PTFP21	47	53	24	47	29	44	56	4/30/2011
PTFP22	56	44	33	45	22	87	13	8/6/2014
PTFP23	56	44	34	45	21	86	14	11/13/2014
PTFP24	57	43	35	45	20	60	40	12/18/2014
PTFP25	71	29	50	40	9	92	6	2/27/2017
PTFP26	59	41	36	47	17	85	15	12/31/2014
PTFP27	58	42	38	41	21	100	0	7/6/2015
PTFP28	71	29	53	37	10	99	1	3/14/2017
PTFP29	76	24	60	33	7	100	0	4/5/2017
PTFP3	38	62	15	47	38	44	56	3/17/2014
PTFP30	59	41	35	48	18	86	9	12/18/2014
PTFP31	59	41	39	42	20	90	10	3/9/2016
PTFP4	60	40	38	44	18	100	0	1/21/2016
PTFP5	71	29	50	43	7	90	0	12/9/2016
PTFP6	63	37	39	48	13	66	34	4/6/2016
PTFP7	57	43	28	57	15	56	44	11/19/2012
PTFP8	60	40	38	45	17	82	17	10/8/2015
PTFP9	68	32	48	40	12	91	9	10/31/2016
mean	59	41	35	48	17	80	19	

Supplementary Table 2. Models tested to account for uncertainty. The original parameters and confidence values are from the ∂a∂i model (5), scaled to the mutation rate ($0.5x10^{-8}$).

Parameters	Original model	CI 95%	Recent split	Old split
		Lower - upper		
NeTX	31,150	27,852 - 34,848	31,150	31,150
NeCFP1	704	56 - 8,404	**56**	**4,202**
NeCFP2	7	NA	7	7
NeCFP3	50	NA	50	50
T_S	3,440	286 - 41,448	**286**	**20,724**

Supplementary Table 3. Average fitness of simulations. In this table we show the average fitness value of simulations for the four models tested and the translocation (T) and no translocation (NT) scenarios for each of them. Each column is the average fitness in the simulated canonical Florida panther (CFP) population. The rows represent different timepoints of the simulation. The rows called x generations, represent the number of generations after the bottleneck.

			Partially recessive dominance effects						Additive dominance effects	
			Original		Old split		Recent split		Original	
	Ne	gen	T	NT	T	NT	T	NT	T	NT
Before bottleneck	*	153896	0.786	0.786	0.797	0.797	0.602	0.602	0.768	0.768
		153897	0.788	0.788	0.797	0.797	0.597	0.597	0.768	0.768
		153898	0.787	0.787	0.797	0.797	0.597	0.597	0.768	0.768
		153899	0.788	0.788	0.797	0.797	0.603	0.603	0.768	0.768
Bottleneck	7	153900	0.788	0.788	0.798	0.798	0.603	0.603	0.767	0.767
		153901	0.788	0.788	0.797	0.797	0.600	0.600	0.768	0.768
		153902	0.794	0.794	0.798	0.798	0.601	0.601	0.760	0.760
		153903	0.736	0.736	0.718	0.718	0.578	0.578	0.756	0.756
		153904	0.701	0.701	0.639	0.639	0.583	0.583	0.752	0.752
		153905	0.665	0.665	0.561	0.561	0.572	0.572	0.740	0.740
		153906	0.641	0.641	0.530	0.530	0.568	0.568	0.743	0.743
		153907	0.625	0.625	0.518	0.518	0.570	0.570	0.752	0.752
		153908	0.602	0.602	0.458	0.458	0.560	0.560	0.748	0.748
		153909	0.577	0.577	0.448	0.448	0.544	0.544	0.744	0.744
		153910	0.562	0.562	0.393	0.393	0.549	0.549	0.742	0.742

Translocation	12	153911	0.543	0.543	0.407	0.407	0.552	0.552	0.743	0.743
		153912	0.637	0.528	0.554	0.402	0.648	0.548	0.763	0.739
	50	153913	0.682	0.528	0.633	0.404	0.679	0.545	0.762	0.736
		153914	0.664	0.543	0.626	0.412	0.679	0.546	0.761	0.739
		153915	0.664	0.544	0.621	0.429	0.663	0.549	0.761	0.736
5 generations		153916	0.655	0.557	0.610	0.435	0.656	0.552	0.763	0.739
		153917	0.652	0.560	0.598	0.449	0.644	0.552	0.760	0.740
		153918	0.646	0.574	0.598	0.456	0.645	0.553	0.760	0.741
		153919	0.637	0.574	0.598	0.464	0.638	0.556	0.762	0.741
		153920	0.633	0.575	0.588	0.469	0.633	0.555	0.763	0.741
10 generations		153921	0.629	0.577	0.594	0.471	0.623	0.556	0.763	0.745
		153922	0.630	0.584	0.580	0.479	0.629	0.556	0.761	0.748
		153923	0.624	0.589	0.582	0.493	0.616	0.555	0.765	0.748
		153924	0.622	0.588	0.576	0.494	0.599	0.556	0.763	0.748
		153925	0.615	0.595	0.577	0.497	0.606	0.558	0.760	0.746
15 generations		153926	0.608	0.592	0.565	0.502	0.605	0.559	0.763	0.750
10 generations		153921	0.629	0.577	0.594	0.471	0.623	0.556	0.763	0.745
50 generations		153961	0.619	0.628	0.591	0.560	0.605	0.564	0.765	0.738
100 generations		154011	0.646	0.627	0.616	0.592	0.614	0.555	0.754	0.738
500 generations		154411	0.583	0.553	0.547	0.513	0.544	0.494	0.673	0.665
1000 generations		154911	0.507	0.467	0.471	0.447	0.465	0.422	0.595	0.604
2000 generations		155910	0.366	0.344	0.330	0.320	0.331	0.300	0.473	0.458
